# 3,5-Dimethyl-2,6-diphenyl-3,4,5,6-tetra­hydro-2*H*-pyran-4-one

**DOI:** 10.1107/S1600536809001792

**Published:** 2009-02-04

**Authors:** Muhammad Nadeem Asghar, Muhammad Nadeem Arshad, Muhammad Zia-ur-Rehman, Islam Ullah Khan, Muhammad Shafiq

**Affiliations:** a Department of Chemistry, Government College University, Lahore 54000, Pakistan.; b Applied Chemistry Research Centre, PCSIR Laboratories Complex, Lahore 54600, Pakistan

## Abstract

The mol­ecular structure of the title compound, C_19_H_20_O_2_, reveals a slightly distorted chair conformation for the tetra­hydro­pyran ring with the two methyl and two phenyl substituents in equatorial positions.

## Related literature

For the isolation of the title compound from its natural source and its biological activity, see: Noller (1966 ▶). For conformational studies, see: Belakhov *et al.* (2002 ▶); Jose Kavitha *et al*. (2003 ▶); Kumar *et al.* (1999 ▶); Ray *et al.* (1998 ▶); Usman *et al.* (2002 ▶). For the synthesis of related molecules, see: Krishnamoorthy *et al.* (2003 ▶); Parthiban *et al.* (2003 ▶).
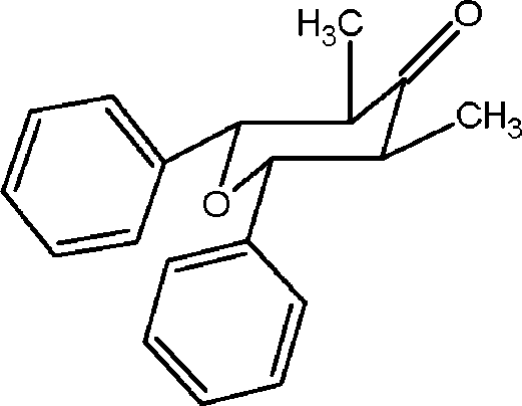


## Experimental

### 

#### Crystal data

C_19_H_20_O_2_
*M* *_r_* = 280.35
Orthorhombic, 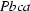

*a* = 14.7247 (10) Å
*b* = 9.2803 (7) Å
*c* = 23.0393 (16) Å
*V* = 3148.3 (4) Å^3^
*Z* = 8
Mo *K*α radiation
μ = 0.08 mm^−1^
*T* = 296 (2) K
0.32 × 0.18 × 0.11 mm

#### Data collection

Bruker APEXII CCD area-detector diffractometer
Absorption correction: none
18754 measured reflections
3885 independent reflections
1969 reflections with *I* > 2σ(*I*)
*R*_int_ = 0.083

#### Refinement

*R*[*F* ^2^ > 2σ(*F* ^2^)] = 0.050
*wR*(*F* ^2^) = 0.163
*S* = 0.92
3885 reflections
190 parameters
H-atom parameters constrained
Δρ_max_ = 0.25 e Å^−3^
Δρ_min_ = −0.20 e Å^−3^

### 

Data collection: *APEX2* (Bruker, 2006 ▶); cell refinement: *SAINT* (Bruker, 2006 ▶); data reduction: *SAINT*; program(s) used to solve structure: *SHELXS97* (Sheldrick, 2008 ▶); program(s) used to refine structure: *SHELXL97* (Sheldrick, 2008 ▶); molecular graphics: *PLATON* (Spek, 2003 ▶); software used to prepare material for publication: *SHELXTL* (Sheldrick, 2008 ▶) and local programs.

## Supplementary Material

Crystal structure: contains datablocks I, global. DOI: 10.1107/S1600536809001792/bt2850sup1.cif

Structure factors: contains datablocks I. DOI: 10.1107/S1600536809001792/bt2850Isup2.hkl

Additional supplementary materials:  crystallographic information; 3D view; checkCIF report

## Figures and Tables

**Fig. 1.**
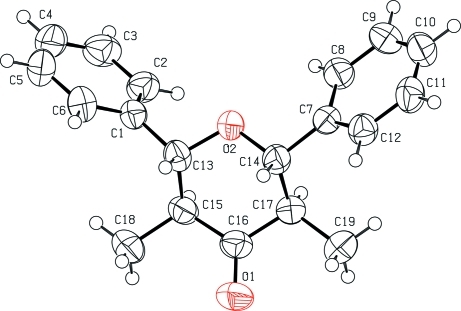
The molecular structure of the title compound, showing 50% probability displacement ellipsoids and the atom-numbering scheme.

## supplementary crystallographic information

### Comment

Tetrahydropyran-4-one moiety has been found in various naturally occurring biologically active heterocyclic compounds (Noller, 1966). Japp and Maitland reported the synthesis of various tetrahydropyrans (Japp & Maitland, 1904) for the first time. Since then, a large number of tetrahydropyran derivatives have been reported with different conformations for the six-membered heterocyclic ring such as sofa (Ray *et al.*, 1998), planar (Kumar *et al.*, 1999), chair (Belakhov *et al.*, 2002; Krishnamoorthy *et al.*, 2003; Jose Kavitha *et al.*, 2003) or twist boat (Usman *et al.*, 2002). Adoption of a particular conformation mainly depends upon the number and nature of the substituents and the level of unsaturation.

In the title compound C_19_H_20_O_2_,as shown in Fig. 1, the tetrahydropyran ring adopts a chair conformation with two methyl and two phenyl groups attached to it. All the methyl and phenyl groups occupy equatorial positions as was also reported for   related molecules (Parthiban *et al.*, 2003; Krishnamoorthy *et al.*, 2003; Jose Kavitha *et al.*, 2003). There are no strong intermolecular interactions between the molecules.

### Experimental

An alcoholic solution of sodium hydroxide (8%; 10.0 ml) was added drop wise to a mixture of 3-pentanone (1.06 ml) and benzaldehyde (2.1 ml) while stirring. The contents were kept stirred for another six hours till the formation of precipitates at bottom which were washed with cold water and re-crystallized in acetone for X-Ray studies.

### Refinement

H atoms bound to C were placed in calculated positions (C—H distance = 0.93 to 0.98Å) using a riding model with U(H) set to 1.2 U_eq_(C) or 1.5 U_eq_(C_methyl_).

### Crystal data

**Table:**

C_19_H_20_O_2_	*F*(000) = 1200
*M**_r_* = 280.35	*D*_x_ = 1.183 Mg m^−^^3^
Orthorhombic, *P**b**c**a*	Mo *K*α radiation, λ = 0.71073 Å
Hall symbol: -P 2ac 2ab	Cell parameters from 1807 reflections
*a* = 14.7247 (10) Å	θ = 2.7–21.7°
*b* = 9.2803 (7) Å	µ = 0.08 mm^−^^1^
*c* = 23.0393 (16) Å	*T* = 296 K
*V* = 3148.3 (4) Å^3^	Needles, orange
*Z* = 8	0.32 × 0.18 × 0.11 mm

### Data collection

**Table:**

Bruker APEXII CCD area-detector diffractometer	1969 reflections with *I* > 2σ(*I*)
Radiation source: fine-focus sealed tube	*R*_int_ = 0.083
graphite	θ_max_ = 28.3°, θ_min_ = 2.2°
Detector resolution: 7.5 pixels mm^-1^	*h* = −12→19
φ and ω scans	*k* = −12→12
18754 measured reflections	*l* = −30→28
3885 independent reflections	

### Refinement

**Table:**

Refinement on *F*^2^	Primary atom site location: structure-invariant direct methods
Least-squares matrix: full	Secondary atom site location: difference Fourier map
*R*[*F*^2^ > 2σ(*F*^2^)] = 0.050	Hydrogen site location: inferred from neighbouring sites
*wR*(*F*^2^) = 0.163	H-atom parameters constrained
*S* = 0.92	*w* = 1/[σ^2^(*F*_o_^2^) + (0.0702*P*)^2^] where *P* = (*F*_o_^2^ + 2*F*_c_^2^)/3
3885 reflections	(Δ/σ)_max_ < 0.001
190 parameters	Δρ_max_ = 0.25 e Å^−^^3^
0 restraints	Δρ_min_ = −0.20 e Å^−^^3^

### Special details

**Table:**

Geometry. All e.s.d.'s (except the e.s.d. in the dihedral angle between two l.s. planes) are estimated using the full covariance matrix. The cell e.s.d.'s are taken into account individually in the estimation of e.s.d.'s in distances, angles and torsion angles; correlations between e.s.d.'s in cell parameters are only used when they are defined by crystal symmetry. An approximate (isotropic) treatment of cell e.s.d.'s is used for estimating e.s.d.'s involving l.s. planes.
Refinement. Refinement of *F*^2^ against ALL reflections. The weighted *R*-factor *wR* and goodness of fit *S* are based on *F*^2^, conventional *R*-factors *R* are based on *F*, with *F* set to zero for negative *F*^2^. The threshold expression of *F*^2^ > σ(*F*^2^) is used only for calculating *R*-factors(gt) *etc*. and is not relevant to the choice of reflections for refinement. *R*-factors based on *F*^2^ are statistically about twice as large as those based on *F*, and *R*- factors based on ALL data will be even larger.

### Fractional atomic coordinates and isotropic or equivalent isotropic displacement parameters (Å^2^)

**Table:**

	*x*	*y*	*z*	*U*_iso_*/*U*_eq_	
O1	0.26436 (11)	0.0831 (2)	0.17630 (7)	0.0820 (6)	
O2	0.49065 (9)	0.29668 (16)	0.21555 (6)	0.0484 (4)	
C1	0.34164 (16)	0.1191 (2)	0.18707 (11)	0.0536 (6)	
C2	0.37801 (14)	0.1225 (2)	0.24820 (9)	0.0509 (6)	
H2	0.4268	0.0509	0.2507	0.061*	
C3	0.42129 (14)	0.2730 (2)	0.25768 (9)	0.0481 (6)	
H3	0.3741	0.3463	0.2524	0.058*	
C4	0.40680 (15)	0.1656 (3)	0.14038 (9)	0.0524 (6)	
H4	0.4528	0.0897	0.1371	0.063*	
C5	0.45719 (15)	0.3048 (3)	0.15765 (9)	0.0493 (6)	
H5	0.4149	0.3860	0.1549	0.059*	
C6	0.46250 (14)	0.2921 (2)	0.31692 (9)	0.0466 (6)	
C7	0.41229 (16)	0.3486 (2)	0.36203 (10)	0.0530 (6)	
H7	0.3527	0.3773	0.3553	0.064*	
C8	0.44825 (17)	0.3634 (3)	0.41681 (11)	0.0622 (7)	
H8	0.4130	0.4013	0.4466	0.075*	
C9	0.53592 (18)	0.3224 (3)	0.42750 (11)	0.0672 (7)	
H9	0.5602	0.3317	0.4646	0.081*	
C10	0.58800 (17)	0.2672 (3)	0.38303 (11)	0.0649 (7)	
H10	0.6479	0.2405	0.3900	0.078*	
C11	0.55156 (15)	0.2515 (3)	0.32831 (10)	0.0562 (6)	
H11	0.5870	0.2133	0.2986	0.067*	
C12	0.53684 (15)	0.3335 (2)	0.11866 (9)	0.0464 (6)	
C13	0.61089 (15)	0.2416 (3)	0.11876 (10)	0.0581 (7)	
H13	0.6120	0.1635	0.1440	0.070*	
C14	0.68301 (16)	0.2649 (3)	0.08169 (13)	0.0701 (8)	
H14	0.7325	0.2026	0.0823	0.084*	
C15	0.68223 (19)	0.3787 (4)	0.04411 (12)	0.0749 (8)	
H15	0.7307	0.3933	0.0189	0.090*	
C16	0.6098 (2)	0.4710 (3)	0.04381 (11)	0.0745 (8)	
H16	0.6094	0.5494	0.0187	0.089*	
C17	0.53748 (17)	0.4482 (3)	0.08070 (10)	0.0607 (7)	
H17	0.4884	0.5112	0.0799	0.073*	
C18	0.30683 (16)	0.0820 (3)	0.29241 (11)	0.0744 (8)	
H18A	0.3330	0.0846	0.3306	0.112*	
H18B	0.2850	−0.0134	0.2844	0.112*	
H18C	0.2573	0.1490	0.2904	0.112*	
C19	0.36211 (17)	0.1792 (3)	0.08100 (11)	0.0801 (9)	
H19A	0.4068	0.2073	0.0528	0.120*	
H19B	0.3151	0.2507	0.0826	0.120*	
H19C	0.3363	0.0881	0.0701	0.120*	

### Atomic displacement parameters (Å^2^)

**Table:**

	*U*^11^	*U*^22^	*U*^33^	*U*^12^	*U*^13^	*U*^23^
O1	0.0478 (10)	0.1251 (17)	0.0730 (12)	−0.0189 (11)	−0.0087 (9)	−0.0002 (11)
O2	0.0483 (9)	0.0578 (9)	0.0389 (9)	−0.0037 (7)	0.0026 (7)	0.0010 (8)
C1	0.0424 (14)	0.0601 (15)	0.0582 (16)	−0.0004 (12)	−0.0031 (12)	−0.0027 (13)
C2	0.0448 (13)	0.0581 (14)	0.0500 (15)	−0.0028 (11)	−0.0009 (12)	0.0031 (12)
C3	0.0447 (12)	0.0545 (14)	0.0450 (14)	0.0039 (11)	0.0017 (11)	0.0024 (12)
C4	0.0433 (14)	0.0677 (17)	0.0462 (14)	−0.0014 (11)	−0.0047 (12)	−0.0027 (12)
C5	0.0501 (14)	0.0552 (14)	0.0427 (14)	0.0081 (11)	−0.0033 (12)	0.0029 (11)
C6	0.0443 (13)	0.0492 (13)	0.0463 (14)	−0.0017 (11)	0.0014 (11)	0.0033 (12)
C7	0.0537 (15)	0.0565 (15)	0.0489 (15)	0.0024 (11)	0.0035 (13)	−0.0002 (12)
C8	0.0755 (18)	0.0663 (17)	0.0447 (15)	−0.0013 (14)	0.0069 (14)	−0.0003 (13)
C9	0.080 (2)	0.0714 (18)	0.0503 (16)	−0.0095 (15)	−0.0124 (16)	0.0052 (14)
C10	0.0531 (16)	0.0769 (19)	0.0646 (18)	0.0011 (13)	−0.0129 (14)	0.0070 (15)
C11	0.0509 (14)	0.0659 (16)	0.0518 (15)	0.0019 (12)	0.0004 (13)	−0.0004 (13)
C12	0.0487 (14)	0.0514 (14)	0.0391 (13)	−0.0006 (11)	−0.0002 (11)	−0.0006 (11)
C13	0.0478 (14)	0.0622 (16)	0.0644 (17)	0.0017 (12)	0.0012 (13)	0.0047 (13)
C14	0.0489 (15)	0.086 (2)	0.076 (2)	0.0030 (14)	0.0021 (15)	−0.0113 (18)
C15	0.0689 (19)	0.105 (2)	0.0512 (17)	−0.0200 (18)	0.0112 (15)	−0.0052 (17)
C16	0.089 (2)	0.083 (2)	0.0518 (17)	−0.0164 (17)	0.0014 (17)	0.0147 (15)
C17	0.0705 (17)	0.0613 (16)	0.0502 (15)	0.0059 (13)	−0.0026 (14)	0.0067 (14)
C18	0.0675 (17)	0.089 (2)	0.0669 (18)	−0.0198 (15)	0.0073 (15)	0.0073 (15)
C19	0.0700 (18)	0.120 (2)	0.0506 (17)	−0.0133 (16)	−0.0127 (14)	−0.0010 (16)

### Geometric parameters (Å, °)

**Table:**

O1—C1	1.212 (2)	C9—H9	0.9300
O2—C5	1.424 (2)	C10—C11	1.378 (3)
O2—C3	1.426 (2)	C10—H10	0.9300
C1—C4	1.504 (3)	C11—H11	0.9300
C1—C2	1.507 (3)	C12—C17	1.377 (3)
C2—C18	1.509 (3)	C12—C13	1.385 (3)
C2—C3	1.550 (3)	C13—C14	1.380 (3)
C2—H2	0.9800	C13—H13	0.9300
C3—C6	1.504 (3)	C14—C15	1.366 (4)
C3—H3	0.9800	C14—H14	0.9300
C4—C19	1.523 (3)	C15—C16	1.367 (4)
C4—C5	1.542 (3)	C15—H15	0.9300
C4—H4	0.9800	C16—C17	1.379 (3)
C5—C12	1.501 (3)	C16—H16	0.9300
C5—H5	0.9800	C17—H17	0.9300
C6—C7	1.379 (3)	C18—H18A	0.9600
C6—C11	1.389 (3)	C18—H18B	0.9600
C7—C8	1.376 (3)	C18—H18C	0.9600
C7—H7	0.9300	C19—H19A	0.9600
C8—C9	1.368 (3)	C19—H19B	0.9600
C8—H8	0.9300	C19—H19C	0.9600
C9—C10	1.378 (3)		
			
C5—O2—C3	113.45 (15)	C8—C9—H9	120.2
O1—C1—C4	122.1 (2)	C10—C9—H9	120.2
O1—C1—C2	122.1 (2)	C11—C10—C9	120.2 (2)
C4—C1—C2	115.83 (18)	C11—C10—H10	119.9
C1—C2—C18	112.26 (18)	C9—C10—H10	119.9
C1—C2—C3	107.24 (17)	C10—C11—C6	120.8 (2)
C18—C2—C3	114.50 (19)	C10—C11—H11	119.6
C1—C2—H2	107.5	C6—C11—H11	119.6
C18—C2—H2	107.5	C17—C12—C13	118.1 (2)
C3—C2—H2	107.5	C17—C12—C5	121.5 (2)
O2—C3—C6	108.08 (16)	C13—C12—C5	120.3 (2)
O2—C3—C2	109.72 (17)	C14—C13—C12	120.6 (2)
C6—C3—C2	113.57 (18)	C14—C13—H13	119.7
O2—C3—H3	108.4	C12—C13—H13	119.7
C6—C3—H3	108.4	C15—C14—C13	120.5 (2)
C2—C3—H3	108.4	C15—C14—H14	119.8
C1—C4—C19	112.98 (19)	C13—C14—H14	119.8
C1—C4—C5	111.25 (18)	C14—C15—C16	119.6 (3)
C19—C4—C5	111.73 (19)	C14—C15—H15	120.2
C1—C4—H4	106.8	C16—C15—H15	120.2
C19—C4—H4	106.8	C15—C16—C17	120.2 (3)
C5—C4—H4	106.8	C15—C16—H16	119.9
O2—C5—C12	107.44 (17)	C17—C16—H16	119.9
O2—C5—C4	111.32 (18)	C12—C17—C16	121.0 (2)
C12—C5—C4	111.73 (18)	C12—C17—H17	119.5
O2—C5—H5	108.8	C16—C17—H17	119.5
C12—C5—H5	108.8	C2—C18—H18A	109.5
C4—C5—H5	108.8	C2—C18—H18B	109.5
C7—C6—C11	117.8 (2)	H18A—C18—H18B	109.5
C7—C6—C3	120.8 (2)	C2—C18—H18C	109.5
C11—C6—C3	121.3 (2)	H18A—C18—H18C	109.5
C8—C7—C6	121.5 (2)	H18B—C18—H18C	109.5
C8—C7—H7	119.2	C4—C19—H19A	109.5
C6—C7—H7	119.2	C4—C19—H19B	109.5
C9—C8—C7	120.0 (2)	H19A—C19—H19B	109.5
C9—C8—H8	120.0	C4—C19—H19C	109.5
C7—C8—H8	120.0	H19A—C19—H19C	109.5
C8—C9—C10	119.6 (2)	H19B—C19—H19C	109.5
			
O1—C1—C2—C18	−2.1 (3)	O2—C3—C6—C11	−34.7 (3)
C4—C1—C2—C18	177.0 (2)	C2—C3—C6—C11	87.3 (3)
O1—C1—C2—C3	−128.7 (2)	C11—C6—C7—C8	−0.6 (3)
C4—C1—C2—C3	50.3 (2)	C3—C6—C7—C8	178.4 (2)
C5—O2—C3—C6	−170.35 (17)	C6—C7—C8—C9	0.3 (4)
C5—O2—C3—C2	65.3 (2)	C7—C8—C9—C10	0.4 (4)
C1—C2—C3—O2	−57.9 (2)	C8—C9—C10—C11	−0.9 (4)
C18—C2—C3—O2	176.88 (18)	C9—C10—C11—C6	0.6 (4)
C1—C2—C3—C6	−178.93 (18)	C7—C6—C11—C10	0.1 (3)
C18—C2—C3—C6	55.8 (3)	C3—C6—C11—C10	−178.8 (2)
O1—C1—C4—C19	6.9 (3)	O2—C5—C12—C17	−125.9 (2)
C2—C1—C4—C19	−172.2 (2)	C4—C5—C12—C17	111.7 (2)
O1—C1—C4—C5	133.5 (2)	O2—C5—C12—C13	56.2 (3)
C2—C1—C4—C5	−45.6 (3)	C4—C5—C12—C13	−66.2 (3)
C3—O2—C5—C12	178.60 (17)	C17—C12—C13—C14	0.0 (3)
C3—O2—C5—C4	−58.8 (2)	C5—C12—C13—C14	177.9 (2)
C1—C4—C5—O2	46.6 (2)	C12—C13—C14—C15	−0.3 (4)
C19—C4—C5—O2	173.93 (18)	C13—C14—C15—C16	0.8 (4)
C1—C4—C5—C12	166.75 (18)	C14—C15—C16—C17	−0.9 (4)
C19—C4—C5—C12	−66.0 (2)	C13—C12—C17—C16	0.0 (3)
O2—C3—C6—C7	146.3 (2)	C5—C12—C17—C16	−178.0 (2)
C2—C3—C6—C7	−91.7 (2)	C15—C16—C17—C12	0.5 (4)
